# The use of wearables for the diagnosis and treatment of Parkinson’s disease

**DOI:** 10.1007/s00702-022-02575-5

**Published:** 2023-01-07

**Authors:** Heinz Reichmann, Lisa Klingelhoefer, Jonas Bendig

**Affiliations:** Department of Neurology, University Hospital Dresden, Fetscherstrasse 74, 01307 Dresden, Germany

**Keywords:** Parkinson’s disease, Telemedicine, Wearables, Sensors, Digital medicine

## Abstract

Parkinson’s disease (PD) is the second most common neurodegenerative disorder, with increasing numbers of affected patients. Many patients lack adequate care due to insufficient specialist neurologists/geriatricians, and older patients experience difficulties traveling far distances to reach their treating physicians. A new option for these obstacles would be telemedicine and wearables. During the last decade, the development of wearable sensors has allowed for the continuous monitoring of bradykinesia and dyskinesia. Meanwhile, other systems can also detect tremors, freezing of gait, and gait problems. The most recently developed systems cover both sides of the body and include smartphone apps where the patients have to register their medication intake and well-being. In turn, the physicians receive advice on changing the patient’s medication and recommendations for additional supportive therapies such as physiotherapy. The use of smartphone apps may also be adapted to detect PD symptoms such as bradykinesia, tremor, voice abnormalities, or changes in facial expression. Such tools can be used for the general population to detect PD early or for known PD patients to detect deterioration. It is noteworthy that most PD patients can use these digital tools. In modern times, wearable sensors and telemedicine open a new window of opportunity for patients with PD that are easy to use and accessible to most of the population.

## Introduction

Over recent decades, the number of patients with neurologic diseases has consistently increased. A typical example is Parkinson’s disease (PD), with about 1 million affected patients (Maserejian et al. 2020) and a worldwide increase of 100% between the years 1990 and 2016 (Ray Dorsey et al. 2018; Rossi et al. 2018; Wanneveich 2018)). With an aging population worldwide, we expect these numbers to increase further in the coming decades. In addition, few countries have enough neurologists and/or geriatricians to guarantee adequate care for PD patients. Furthermore, many parts of the world are rural, with limited access to movement disorder specialists, forcing many patients to travel long distances to find adequate care. The latter is a problem for PD patients since, in the advanced stages, they cannot easily travel and need support. In Germany, Europe, and the United States, more than 40% of PD patients are treated by general practitioners, not by neurologists, and certainly not by movement disorders specialists. Most of us use questionnaires and take the patient’s history to evaluate and compare motor and non-motor symptoms between visits. Unfortunately, it is well known that the patients’ reports are subjective (Ossig et al. 2016) and differ from objective measurements. Studies show that even in the long course of PD, patients have problems differentiating between specific PD symptoms, such as tremors and dyskinesia, and between motor and non-motor fluctuations (Papapetropoulos 2012; Goetz et al. 1997). In addition, the reports depend on the patient’s mood at the consultation time. Thus, if the patient is feeling fine, they report positive since the last consultation, but if the patient’s mood is bad, the previous months are often reported as negative. Frequently the caregiver will intervene and put things into perspective: This is also an issue for for trials conducted to test new therapies or new symptom coverage. The assessment of mild, fluctuating symptoms and their development over an extended period is challenging to recall by patients, but on the other hand, it is of high scientific and methodological interest. Some studies previously revealed negative results, possibly due to the rough instruments used to measure outcomes, which would not detect slight changes (Maetzler et al. 2009; Maetzler et al. 2013; Marker and I. 2011). Therefore, wearables offer the chance to remotely measure symptoms objectively over time and thus detect small changes. All of these are important considerations for new ways/possibilities in patient care. Thus we believe that we should use modern approaches in the “digital era” to help serve as objective measures.

In this article, which does not claim to be complete, we want to underline our view that there are already excellent digital tools to support patient care. The majority of studies conducted so far used wearable sensors or smartphone apps. Especially during the COVID-pandemic, telemedicine has become increasingly popular and has emerged as a new field in monitoring and counseling more PD patients.

Wearables are more objective than diaries and allow the clinician to subtract needed information, overcoming the problem of elderly patients forgetting or misinterpreting motor and non-motor symptoms associated with PD. Often they cannot recall what happened during the last few weeks resulting in clinicians having difficulty giving good advice, especially when time is restricted, as is the case in most neurologic in-and-out patient clinics. In Germany, neurologists are paid for only four consultations per year for each patient with PD. This does not support a continuous exchange with patients and indicates that we have to be open to new solutions.

### The use of wearables and smartphone apps for early detection of PD

A short review of smartphones for remote symptom monitoring in PD reports that the development of such tools was first published in 2013 (Little 2021).

We have developed an app for the early detection of PD, which was supported by a European Horizon 2020 grant given to centers in Greece, Portugal, the U.K., Germany, Sweden, and Belgium. We called the app “iPrognosis” (Iakovakis et al. 2019, 2020, 2022; Hadjidimitriou, et al. 2016; Klingelhoefer, et al. 2017; Klingelhoefer, et al. 2019; Klingelhoefer, et al. 2019; Laganas et al. 2021), and it can be downloaded from the google play store on any android smartphone for free by healthy volunteers or patients aged 40–90 years of age with early PD. The iPrognosis app is only active after the written, informed consent for study participation within the app and the provision of a few essential items of personal information such as age, gender, personal health status (whether healthy or diagnosed with PD), and family history (whether PD is known in the family). The app also recorded years of formal education and familiarity with smartphones. These data were necessary to aid in the assignment of the remotely collected data on the handling of the smartphone to either healthy controls or PD patients since the study participants were unknown to the medical centers participating. Furthermore, the study participants could define within the app whether they wanted to be informed if any abnormalities were observed or not.

**Fig. 1:**
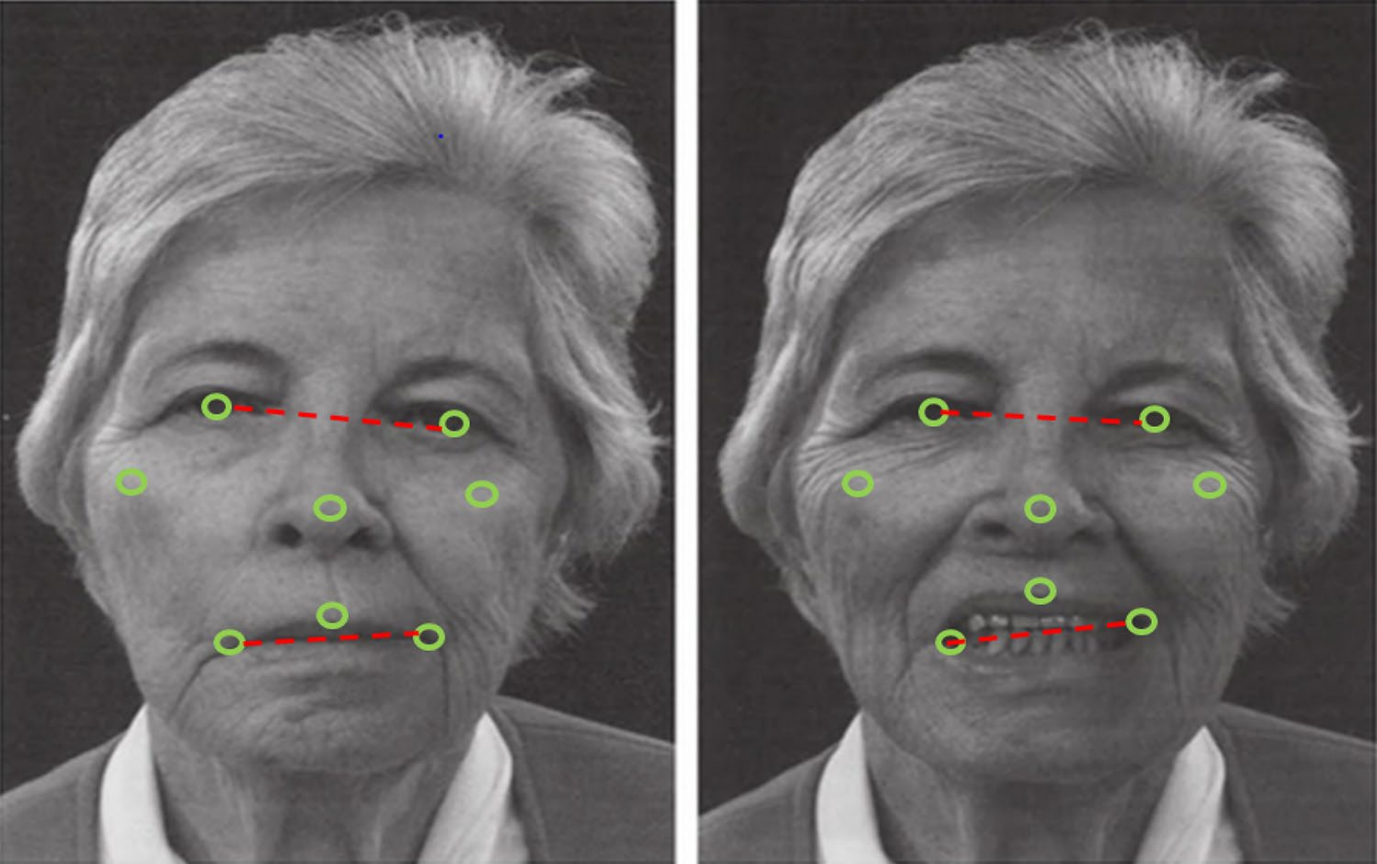
Facial expression-based model of the iPrognosis app for detection of hypomimia and depression

The iPrognosis app measured the general usage data of the study participants when interacting with their smartphones in a way that neither interfered with nor influenced the smartphone users in their daily activities. We investigated whether the iPrognosis app can recognize patterns corresponding to the motor and non-motor symptoms of PD. For this purpose, we investigated the following parameters: speech, movement, dynamics of keyboard use, facial expression in selfies, and emotional content in text messages (Klingelhoefer, et al. 2017, 2019, 2019). If the patient made a phone call via the smartphone, the app did not capture the content, but the app analyzed the patient’s voice to detect dysarthrophonia and speech festination. The content of messages written on the iPrognosis keyboard was also not recorded, but the dynamics of keyboard use, such as flight time, pressure, and hold time of the different keys, were registered to detect brady- and hypokinesia, tremor, and rigidity. Thus, if a member of the study cohort who used the iPrognosis app got slower in writing (due to tremor) or made irregular, double, or triple clicks, this was registered. Hypomimia and depression were analyzed when patients took selfies. Again, while the app monitored the facial expression, no pictures left the smartphone. Using 12 defined points in the face (eyes, nose, mouth, cheeks, ears), the patient’s smile, facial asymmetry, and whether their eyes or mouth were open were used to investigate hypomimia or even depression (Fig. 1). Similarly, the app investigated emotions and moods by analyses of text messages based on a coding scheme without knowing the content.

The iPrognosis app was available on the google play store from May 2017 for at least four years, depending on the different ethical approvals. All data collected was anonymized on the smartphone before being automatically uploaded to the cloud. The iPrognosis app and the corresponding adaptive computer algorithms for recognizing PD suspicious symptoms and any change of symptoms/behavior over time were optimized with increasing data collection. Furthermore, study participants who agreed to be contacted were invited by a pop-up message for a medical examination by a movement disorders specialist in one of the three medical centers of the project.

At the end of the study, 4486 people had downloaded the iPrognosis app, and 2624 people consented to study participation with a mean age of 49 ± 16 years, 63% being male, 84% declared to be healthy, and 16% diagnosed with PD (Klingelhoefer et al. 2019). From the 1529 study participants who agreed to be contacted, 112 were investigated for motor and non-motor symptoms of PD by a movement disorders specialist and by instrumental diagnostics such as olfaction test, substantia nigra sonography, etc. in one of the three participating medical centers (Klingelhoefer et al. 2019). Ninety-seven followed up after 0.58 ± 0.03 years (median, standard error) for symptom changes. The medical evaluation of the study participants by an expert with differentiation in PD patients or healthy controls was the gold standard. The mean duration of PD of 4.23 years, ranging from 0.38 up to a maximum of 10.46 years of PD, corresponded well with patients at an early stage of the disease (Chen et al. 2016; Schuepbach et al. 2013). This was further underlined by the mean duration of 3.92 years of PD medication intake. In addition, 97% of PD patients were evaluated to be in a stable “ON” condition during the study assessment at baseline and 94% at the follow-up assessment. Six PD patients were drug naïve throughout the first and follow-up assessment, whereas in one drug naïve PD patient, dopaminergic medication was commenced. One healthy participant was diagnosed with PD during the assessment period, and PD medication was initiated during the follow-up assessment.

We must note that the study participants could select what type of data, e.g., speech, movement, text messages, etc., they wanted to record through the iPrognosis app leading to an imbalance in the volume of app recordings for each data type. Additionally, the volume of data per participant depends on the frequency of use of their smartphone devices. In the medically evaluated study (participants *n* = 112), we compared the results of the different iPrognosis app-based recordings for each data type with the corresponding clinical item, e.g., for voice degradation, we compared iPrognosis app voice-based model with UPDRS Part III Item 18. For tremors, the iPrognosis app IMU-based indicator (Papadopoulos et al. 2019) was compared with the UPDRS part III items 20 and 21, while iPrognosis app typing pattern-based models were compared with UPDRS part III item 22 (rigidity), 23 (finger tapping), 31 (body bradykinesia/ hypokinesia), to allow calculation of diagnostic properties. Furthermore, these results were compared with those of the study participants who have had purely remote self-reported health states (*n* = 1529).

The results of the discrimination performance (PD patients versus healthy people) for the different data types are presented in Table 1 (Klingelhoefer et al. 2019, 2019). We achieved the best results with a high classification performance for both medically assessed study participants and remote purely self-reporting study participants for the IMU-based module for tremor detection (Papadopoulos et al. 2019, 2019) and the data-based typing models for bradykinesia and rigidity detection (Iakovakis et al. 2019; Iakovakis et al. 2018). The typing database models were developed further so that, finally, for three smartphone app indicators, the same diagnostic accuracy for differentiation between PD patients and healthy people could be reached compared to a UPDRS-based evaluation by a movement disorders specialist (Iakovakis et al. 2020).

**Table 1:** Diagnostic performance of the iPrognosis app-based models of the different data types

Data types	Diagnostic Accuracy for differentiation in	
	Medically-evaluated PD patients and Healthy Controls	Self-reported PD patients and Healthy Controls
Voice-based models for detection of dysarthrophonia	0.68	0.58
Tremor-based models for detection of rest and action tremor	0.96	0.87
Typing-based models for detection of		
* Rigidity*	0.87	0.75
* Finger Tapping*	0.86	0.74
* Body Bradykinesia/Hypokinesia*	0.83	0.73
Facial expression-based models for detection of hypomimia	0.72	0.61

For the differentiation of de-novo, drug-naive PD patients, and healthy people, the diagnostic accuracy for all three smartphone app indicators was even better than for the UPDRS-based evaluation (Iakovakis et al. 2020). Unfortunately, the voice-based model, intended to discriminate between PD patients and healthy controls based on voice samples/features recorded and extracted during phone calls, yielded moderate to low classification performance. This may be attributed, to a certain extent, to the nature of the raw data, i.e., voice samples captured in an uncontrolled environment with various ambient noise levels, etc., as well as the lack of raw data (due to privacy reasons) that would have allowed for more elaborate noise-reduction techniques to be applied. Based on the iPrognosis implementation, raw voice samples are processed locally on the smartphone, and only features are uploaded to the Cloud and made available to researchers.

Differentiation between PD patients and healthy people based on the assessed non-motor symptom (NMS) such as the text (SMS)-based depression detection model as well as the model that relies on front-facing camera photos (‘selfies’) for detecting hypomimia, did not reach relevant diagnostic accuracy as single indicators. This was mainly related to the low sample size of participants who allowed the relevant data to be collected. Taken together, we could define parameters in the iPrognosis app that can be measured via smartphone, yielding a high diagnostic performance to detect patients with PD.

### The use of wearables (sensors)

As outlined in Table 2, several well-developed systems for symptom coverage in PD exist. Not all of them provide continuous monitoring over a given time; some do not cover symptoms such as freezing of gait, gait abnormalities, or postural instability. They provide all basic information on bradykinesia and dyskinesia, however.

**Table 2:** Overview on wearables for Parkinson’s disease

Company	Symptoms covered	Continuous monitoring during use
Great Lake Neurotechnologies	Tremor, Bradykinesia, Dyskinesia	No
Great Lake Neurotechnologies	Tremor, Bradykinesia, Dyskinesia	Yes
Clear Sky	Dyskinesia	No
Clear Sky	Motor fluctuations, bradykinesia, FoG	No
Global kinetics	Bradykinesia, Dyskinesia, Tremor	No (24 h over 7 days)
Sense 4 Care	Motor fluctuations, Bradykinesia, FoG, Gait	Yes
PD Neurotechnologies	Bradykinesia, Dyskinesia, Tremor, FoG, Gait, postural Instability, motor fluctuations	Yes

Bold characters discussed in the review

*FoG* Freezing of gait

A rather comprehensive overview of other wearables used in the home-based assessment of abnormal movements in PD is given by Ancona et al. (Ancona et al. 2022).

Our group has worked with Global Kinetics and PD Neurotechnologies and thus gained experience with these tools. Other reviews describe some of the commonly used wearables [e.g., Monje et al. (2019), Channa et al. (2020), Sweeney et al. (2019), Hansen et al. (2018), Ramsperger et al. 2016, Rovini et al. (2017)].

**Fig. 2:**
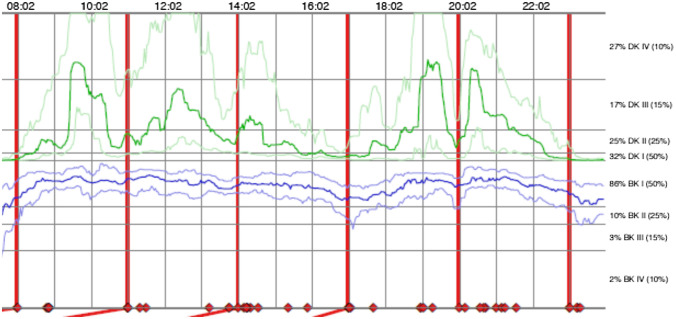
Section of a PKG report summarizing the seven day recording from 8AM till 11 PM of a PD patient with severe dyskinesia—peak dose (around 9:30AM, 12:30AM) and end off dose dyskinesia (around 7PM) as shown by the fat green line and mild bradykinesia throughout the day with slight worsening around 5PM and 11 PM as shown by the fat blue line. The red lines present the determined Levodopa intake times and the red quadrats the confirmed medication intake time of the PD patient

The Parkinson’s KinetiGraph (PKG) is a wrist-worn data logger which uses accelerometry to collect movement data (Griffiths et al. 2012). Generally, this watch-like sensor is worn on the wrist on the more affected side and scores every two minutes by providing simultaneous measures of bradykinesia and dyskinesia (Fig. 2). Furthermore, it can detect tremors. Immobility and removal of the device from the wrist are reported, and patients’ parameters are compared to the motor function of a healthy control group (Farzanehfar et al. 2022).

We used this tool to monitor nighttime sleep in PD (Farzanehfar et al. 2022). PD patients with excessive daytime sleepiness (EDS) (ESS > 12 points, PD-EDS group, *N* = 30) were compared to “non-sleepy” PD patients (ESS < 10 points, PD-NS group, *N* = 33) (Klingelhoefer et al. 2016). Basic and disease-related information, and motor and non-motor assessments were obtained in the clinic. At home, PKG recordings were obtained on the more affected wrist for six consecutive 24 h periods, while Hauser diaries were filled out over the same period. We developed indicators for nighttime sleep quantity and quality of the PKG, and for nighttime sleep quantity of the Hauser diaries. We defined these indicators using state-of-the-art methodologies, specific actigraphy for nighttime sleep assessment, and based on the investigation of immobility measurement by PKG in association with daytime polysomnography (Klingelhoefer et al. 2016; Kotschet et al. 2014). Our study revealed an impressive and noticeable difference between PD patients with and without good nighttime sleep, as assessed by PKG. In PD patients with EDS, the results of validated questionnaires and scales evaluating nighttime sleep disturbances such as insomnia, parasomnia with nocturnal restlessness (as a sign of restless legs syndrome), nightmares and hallucinations, etc., correlated significantly on a moderate to high level with the PKG’s parameter for quantity and quality of nighttime sleep. This was not found in PD patients without EDS. No significant correlation between the nighttime sleep quantity parameters of the Hauser diary with subjective sleep perception was found in the PD-EDS or the PD-NS group. On the contrary, the PKG systems could nicely differentiate between PD patients with disturbed nighttime sleep and those without disturbed nighttime sleep. In addition, it provided information about nighttime sleep quantity and quality. It helped to differentiate between insomnia, parasomnia, restless legs syndrome, and RBD.

Based on this knowledge, we further investigated whether monitoring nighttime sleep is possible with a smartwatch app (Iakovakis et al. 2020). Smartwatches contain accelerometers and gyroscopes, similar to actigraphy or the PKG. We investigated early-stage PD patients and age-matched healthy controls using validated questionnaires and scales for nighttime sleep in the clinic, following which, nighttime sleep was monitored in all study participants at home over the course of 3 months, using a self-developed smartwatch app. Additionally, polysomnography (PSG), the gold standard, was performed in parallel with the smartwatch-based monitoring in some study participants. When comparing the smartwatch-based monitoring and the PSG monitoring of nighttime sleep, we determined that the smartwatch app could assess indicators of nighttime sleep quantity and quality such as sleep latency, the total duration of sleep, and the efficacy and fragmentation of sleep. Further, based on the smartwatch app-derived data, it was possible to differentiate between early-stage PD patients and healthy controls (Iakovakis et al. 2020).

The gut is one of the first organs affected by PD-specific pathology, and gastrointestinal symptoms, which appear in prodromal PD and throughout the course of the disease, are the most common NMS. Therefore, in collaboration with specialists on eating behavior at the Karolinska Institute in Sweden, we investigated differences in objectively measured energy intake between healthy controls (HC), and early and advanced-stage PD patients, during a standardized lunch in a clinical setting (Fagerberg, et al. 2019, 2020) in a prospective, cross-sectional study. We hypothesized that both the cardinal motor symptoms of PD, which impact the fine motor skills involved in handling the cutlery, and NMS such as olfaction, taste, and rate of passage of food throughout the intestine, have an impact on eating behavior and the energy balance (Fagerberg, et al. 2019, 2020). Furthermore, we investigated whether these changes in eating behavior in PD patients can be assessed and monitored during a meal with a smartwatch-based app (Fagerberg, et al. 2019, 2020). Thus, we introduced a Plate-to-Mouth indicator, measured by a smartwatch app worn on the dominant hand that measures said hand’s time spent operating a utensil to transfer a quantity of food from the plate into the mouth during the course of a meal (Kyritsis et al. 2021). With this objective eating behavior indicator, we revealed an Area Under the Curve (AUC) of 0.748 for a controlled clinical dataset and 0.775/1.000 for two remote datasets toward the classification of in-meal eating behavior profiles to the PD or HC group (Kyritsis, et al. 2020). In addition, the advanced-stage PD patients had a significantly lower energy intake compared with HC (− 162 kcal, *p* < 0.05) and compared with early-stage PD patients (− 203 kcal, *p* < 0.01). The number of spoonfuls consumed, eating problems, dysphagia and upper extremity tremor could explain most (86%) of the lower energy intake in advanced stage PD vs. HC, while the first three of these could explain around 50% of the lower energy intake in advanced compared to early-stage PD patients (Fagerberg, et al. 2019). In summary, we were able to demonstrate that it is possible, using a smartwatch app, to perform objective, remote monitoring of eating behavior and to differentiate between PD patients and HC. This is important, since reduced energy intake, as observed especially in advanced-stage PD patients, is associated with weight loss, malnutrition, and overall higher morbidity.

The so-called STAT-ON (Company Sense4Care) device was developed in Spain over the last 12 years. This sensor, worn around the waist, allows gait analysis, and monitoring of bradykinesia, dyskinesia, freezing of gait, the detection of falls, and analysis of movement quantity (Rodriguez-Martin et al. 2022). It gained its CE in June 2019. The device was rated highly by physicians for detecting PD symptoms, and there was a high level of satisfaction with the device in PD patients and their caregivers (Rodriguez-Martin et al. 2020). Several studies are underway to test for the correlation between the findings obtained from regular clinical observation and the device, and clinical studies in which the device will be used (Rodriguez-Martin et al. 2022).

An even more comprehensive evaluation of bradykinesia, dyskinesia, tremor, freezing of gait, postural instability, and motor fluctuations can be gained by use of the PDMonitor™ Monitoring System (Tsiouris et al. 2017). These wearable sensors are worn on the waist, both wrists, and both legs. Each device collects movement data using an accelerometer, a gyroscope, and a magnetometer and allows the recording of both sides of the body of a PD patient. In addition, there is a PDMonitor™ mobile app, which can be used to register the intake of medication or send questions from the patient or caregiver to the treating physician. Information from the sensors and the app is transferred to a cloud and can be accessed by the physician via the PDMonitor™ physician tool. The physician tool provides, via the mobile app, a summary of the motor symptoms and mobility status of the patient, complemented by information about medication and nutrition. Due to the instructive outline of the physician tool, an excellent interpretation of various forms of dyskinesia can be made, and the motor status of patients can be compared with diaries. Due to the continuous registration, the effectiveness of a given anti-Parkinson medication can be monitored throughout the whole day, and adequate adjustments can be made if the patient is not in the therapeutic window.

Wearables and apps need some basic knowledge of modern technology. Since our center is in the process of establishing a special network for patients with advanced PD in our area, we were interested to find out if elderly patients possessed the required know-how (Bendig et al. 2022). We selected 12 patients and utilized video visits, a smartphone app, a camera system in their homes, and wearable sensors over an observation period of 12 weeks. Patients were familiarized with the devices and procedures through group-based online training and a training session at home. The usability of our tools for treating patients via telemedicine was tested with questionnaires and semi-standardized telephone interviews. The mean age of our patients was 65 years with a mean disease duration of 7 years. None of the patients had a cognitive decline. During the observation period, we initiated 27 therapeutic adjustments of which 7 consisted of physiotherapy, occupational therapy, and speech-language therapy. There was a good agreement between our camera visits and the recording by the PDMonitor™ system. On average, with the exception of filling out an electronic version of the Hauser diary and confirming the medication on the smartphone, there was good adherence to the different devices. Patients frequently contacted our team with technical problems (*n* = 20), especially in the first two weeks of the study, and in total there were more patient-initiated contacts related to technical issues (*n* = 39) than medical issues (*n* = 27). Overall, patients were very satisfied with the systems, the technical support, and the medical treatment during the study. We concluded that the majority of the elderly did well, but that digital technologies are not suited to all patients. Another important conclusion from this study was that close contact with a support coordinator and the treating physician is crucial. With this setup, our patients could avoid traveling over long distances, and we were able to adjust treatment via telemedicine.

In summary, wearables and digital technologies are required in the evaluation and treatment of Parkinson’s disease, because in most countries there are not enough experienced physicians relative to the increasing number of PD patients. In addition, this elderly population is unable to travel long distances to specialized neurological centers, and few countries have specialized nurses able to travel to the patient’s homes. In our view, movement disorder specialists should introduce telemedicine visits to satisfy this need. Telemedicine and wearables will be a bridge between the specialist center and the patients. Apps may help us to identify patients at risk of developing PD and to detect PD patients with worsening motor and non-motor symptoms. Modern wearables can at least detect bradykinesia, dyskinesia, and motor complications. In addition, modern technology will allow the teaching of patients, and enable the exchange of data and questions between the patient and the treating physician. The COVID-pandemic has proven that our patients accept such modern technologies and that these open a new window of opportunity to deal with their challenges.

## Data Availability

Compare with original literature. The datasets used and/or analyzed for this study could be obtained from the corresponding authors cited in this paper.

## Abbreviations

*EDS*: Excessive daytime sleepiness
*HC*: Healthy control
NMS: Non-motor symptoms
*NS*: Non-sleepy
*PD*: Parkinson’s disease
*PKG*: Parkinson’s Kinetigraph
*UPDRS*: Unified Parkinson Disease Rating Scale
